# Lipid Adjustment in the Analysis of Environmental Contaminants and Human Health Risks

**DOI:** 10.1289/ehp.7640

**Published:** 2005-03-17

**Authors:** Enrique F. Schisterman, Brian W. Whitcomb, Germaine M. Buck Louis, Thomas A. Louis

**Affiliations:** ^1^Division of Epidemiology, Statistics and Prevention Research, National Institute of Child Health and Human Development, National Institutes of Health, Department of Health and Human Services, Rockville, Maryland, USA; ^2^Department of Biostatistics, Johns Hopkins Bloomberg School of Public Health, Johns Hopkins University, Baltimore, Maryland, USA

**Keywords:** causal modeling, directed acyclic graphs, organochlorines, polychlorinated biphenyls, risk estimation, serum lipids

## Abstract

The literature on exposure to lipophilic agents such as polychlorinated biphenyls (PCBs) is conflicting, posing challenges for the interpretation of potential human health risks. Laboratory variation in quantifying PCBs may account for some of the conflicting study results. For example, for quantification purposes, blood is often used as a proxy for adipose tissue, which makes it necessary to model serum lipids when assessing health risks of PCBs. Using a simulation study, we evaluated four statistical models (unadjusted, standardized, adjusted, and two-stage) for the analysis of PCB exposure, serum lipids, and health outcome risk (breast cancer). We applied eight candidate true causal scenarios, depicted by directed acyclic graphs, to illustrate the ramifications of misspecification of underlying assumptions when interpreting results. Statistical models that deviated from underlying causal assumptions generated biased results. Lipid standardization, or the division of serum concentrations by serum lipids, was observed to be highly prone to bias. We conclude that investigators must consider biology, biologic medium (e.g., nonfasting blood samples), laboratory measurement, and other underlying modeling assumptions when devising a statistical plan for assessing health outcomes in relation to environmental exposures.

Persistent lipophilic xenobiotics pose particular methodologic challenges when assessing potential human health risks. The human health effects literature on exposure to lipophilic agents such as organochlorines (OCs) is equivocal, impairing our ability to quantify risks (Calle et al. 2002; Hunter et al. 1997; Laden et al. 2001a, 2001b). For example, Wolff and colleagues (Wolff 1985; Wolff and Toniolo 1995; Wolff et al. 1993, 2000) found an increased odds ratio for breast cancer for the highest quintile of wet-weight dichlorodiphenyl-dichloroethylene (DDE) and polychlorinated biphenyls (PCBs; expressed as nanograms analyte per milliliter serum) when compared with the lowest quintile, whereas Laden et al. (2001a, 2001b) found no association when concentrations of DDE and PCBs were standardized for serum triglycerides and cholesterol. No association was reported for PCBs and risk of breast cancer when expressing concentrations either as wet weight or lipid standardization values (Helzlsouer et al. 1999).

Varying laboratory practices for expressing PCB concentrations may in part account for the equivocal findings for human health end points. Serum PCB concentrations, as with other lipophilic xenobiotics, are dependent on serum lipid concentrations (Eyster et al. 1983; Guo et al. 1987). Under certain circumstances an equilibrium is reached, and information regarding serum PCB levels and serum lipid levels may be predictive of PCB body burden (Brown and Lawton 1984). If serum lipids indeed act in this manner, higher serum lipid levels should correspond to higher serum PCB concentrations (Calvert et al. 1996). However, serum OC concentrations and lipids are affected postprandially and need to be considered in relation to quantity and timing of food consumption (Phillips et al. 1989). When it is not possible to collect adipose tissue, serum samples are frequently used. However, serum (or plasma) introduces methodologic challenges with regard to lipids when estimating health risks, particularly when nonfasting samples are used (Whitcomb et al. 2005). Collection of fasting samples can hamper the feasibility of epidemiologic research and may adversely impact study participation. Nonfasting samples require further attention to serum lipids (Brown and Lawton 1984; Brown et al. 1994; Eyster et al. 1983).

Our limited understanding of the true relation between serum and adipose tissue concentrations of lipophilic xenobiotics in relation to serum lipids and particular health outcomes makes model specification difficult (Calvert et al. 1996; Mussalo-Rauhamaa 1991). Investigators typically express measurements on a wet-weight basis or per unit volume of serum or as lipid-standardized values, where the concentration is divided by serum lipids.

Lipid standardization may be useful for comparing exposure concentrations across tissue specimens or across study populations by expressing PCB concentrations per gram of fat (Morgan and Roan 1970). Use of lipid weight (PCB per unit of serum lipids) as opposed to wet weight (PCB per unit of serum) has been advocated for the measurement of persistent lipophilic chemicals (Brown and Lawton 1984), especially if one assumes body burden equilibrium. Other approaches reported in the literature include the use of a log-linear model with serum lipids included as a separate term in the regression equation (Moysich et al. 1998). Other investigators have conducted two-stage analyses wherein serum lipids are regressed on serum PCB concentrations with the residuals entered as an individual risk factor (Hunter et al. 1997).

The issue of how best to model the relation among serum PCBs, lipids, and health outcomes remains an understudied area critical for the assessment of health effects. Here we demonstrate the impact of model (mis) specification and its effect on the interpretation of study findings. We used directed acyclic graphs (DAGs) to define a causal framework among exposure, lipids, and health outcome and values for parameters as informed by the literature (Hernan et al. 2002; Robins et al. 2000). Using DAGs to supply a causal framework and parameter values informed by the literature, we present the results of a simulation study. These results identify the best statistical model for each circumstance and the bias produced by a mismatch between the DAG and the statistical analysis.

## Materials and Methods

### Statistical models and DAGs.

Optimal modeling of the statistical relations among serum PCBs, serum lipids, and health outcomes requires positing an underlying causal model that reflects the following considerations: *a*) biologic plausibility; *b*) laboratory capability for quantifying compounds and lipids; *c*) underlying statistical assumptions (e.g., error structure); and *d*) other relevant study covariates (e.g., known and potential confounders). To focus on bias, we assume perfect laboratory measurement of PCBs and the absence of unmeasured confounding.

We depict each scenario via a simple DAG that shows relations but does not dictate a specific statistical model (i.e., mean and error structures). A single-headed arrow represents a causal relation between the ancestor (tail) and the descendant (head). A dashed line represents a noncausal association between two variables, suggesting a shared ancestor that may or may not have been measured; the absence of an arrow signifies no relation.

The true causal structure relating PCBs and serum lipids depends on the outcome under study. Investigators typically have insufficient biologic information to specify the correct analytic model, often resulting in analytic strategies based on unverified assumptions. For example, research indicates a possible causal effect of PCBs on serum lipid levels (Hennig et al. 2005; Langer et al. 2003). Additionally, lipid levels have been suggested to affect breast cancer risk (Atalay et al. 2004), but their impact on other health end points has received limited attention. For our purposes in this study, our scenarios, hypothetical “causal truths,” are based on the literature and their relation to frequently used statistical models.

### Statistical models.

We investigated four statistical models (unadjusted, standardized, adjusted, and two-stage) for the analysis of hypothesized PCB exposure, serum lipids, and a health outcome (breast cancer), along with eight plausible DAGs for each model to illustrate the choices facing investigators. For illustrative purposes, all models assume that there are no unmeasured confounders. For all models, *P* = *Pr*(*Y* = 1|*X*, *SL*), where *Y* is a dichotomous dependent variable representing the presence/absence of the disease; *X* = PCB; and *SL* = serum lipids.

#### Unadjusted model.

The unadjusted model is equivalent to the use of wet-weight values when estimating the effect of an exposure such as PCBs on a health outcome without further consideration of serum lipids.


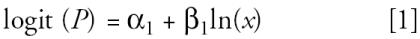


**Figure 1:**
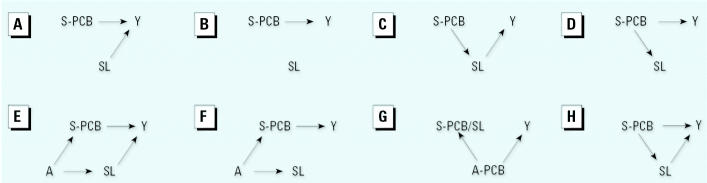
Causal scenarios for relations among PCB, serum lipids (SL), and outcome (*Y*). (*A*) PCB and SL are marginally dependent conditional on *Y*; serum PCB (S-PCB) causes *Y*, and SL causes *Y*. (*B*) PCB as cause of *Y*; S-PCB causes *Y,* independent of SL. (*C*) PCB and *Y* are marginally dependent on and blocked by SL; S-PCB causes SL, which causes *Y.* (*D*) *Y* and SL are marginally dependent and blocked by serum PCB; S-PCB causes *Y* and SL. (*E*) PCB and SL are marginally dependent conditional on both the shared ancestor variable, *A*, and *Y*. An unmeasured variable, *A*, causes both S-PCB and SL, each of which independently causes the outcome; this is the traditional situation of confounding, with SL acting as a confounder of the relation between serum PCBs, PCBs, and *Y.* (*F*) PCB and SL are marginally dependent on the ancestor, *A*; SL and *Y* are marginally dependent on A and, thus effectively, on PCB. S-PCB and SL are caused by *A*, but only PCB is causally related to *Y.* (*G*) PCB per unit SL and *Y* are marginally dependent conditional on adipose tissue PCB. Adipose tissue PCB (A-PCB) causes serum PCB per unit serum lipid and causes *Y;* PCB and outcome are correlated rather than directly causally related. (*H*) Blocked and unblocked path. *Y* is both directly caused by PCB and marginally dependent conditional on SL; S-PCB causes *Y,* as well as SL, which causes *Y*.

Accordingly, this model is suitable for use when it is reasonable to assume that serum lipids are not a confounder. This assumption holds true regardless of the relation between lipids and the outcome. Inclusion or exclusion of lipids as an adjustor may affect model fit, but it will not impact PCB exposure/response estimates. Four DAGs, shown in Figure 1, are appropriately evaluated by use of the unadjusted statistical model. Figure 1A reflects a scenario that will result in an unbiased risk estimate as serum lipids are assumed to be unrelated to PCB levels. Use of this model for Figure 1B yields optimal estimates, if serum lipids are unrelated to both PCBs and the outcome.

An unadjusted model is also appropriate for Figure 1C, where PCBs are assumed to have an indirect effect via serum lipids; adjustment for a variable in the causal pathway may introduce an undesirable bias when estimating direct effects (Greenland 1996, 2003; Greenland and Morgenstern 2001).

In Figure 1D, PCBs are assumed to affect both serum lipids and the outcome, creating a spurious association (Robins et al. 2000). Here, only an unadjusted model is appropriate for risk estimation. Because they vary with PCBs, adjustment for serum lipids is tantamount to partial adjustment for the exposure itself.

#### Standardized model.

The lipid-standardized model is one way to account for the effect of serum lipids on serum PCB levels. This model is used frequently and is conceptually similar to use of the body mass index (BMI; weight in kilograms divided by the squared height in meters) to adjust weight for height in measuring adiposity.


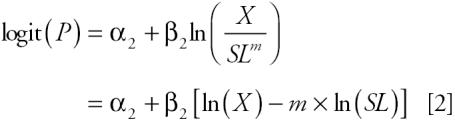


The power, *m* in Equation 2 is a factor that generalizes the relation of PCBs and serum lipids. Due to measurement error in the quantification of lipids, use of Equation 2 when Figure 1A holds can result in biased estimates. If Figure 1B holds, estimates will be affected by a scaling issue, as the beta coefficient is that for the log of the ratio of PCB to lipids. If the true relations follow Figure 1 (C or D), then use of Equation 2 will adjust, albeit incompletely, for the exposure of interest, as in both Figure 1C and D, PCBs determine the variance of serum lipids. Figure 1C depicts a causal relation between both PCBs and serum lipids with the outcome, and a noncausal association between PCBs and serum lipids resulting from a common ancestor, A. Use of the standardization model will be valid for this situation only if the standardization completely accounts for the association between PCB and serum lipids. Otherwise, use of this model will result in biased estimates.

Figure 1F is modeled similarly to Figure 1D in that the relation between PCBs and lipids is due to a common cause, A. In this scenario, the standardized model again suffers from a scale issue. All other models will produce unbiased estimates, but precision of the estimate may vary depending on several factors, including measurement error. The potential error associated with the measurement of serum lipids can exceed that for the analyte itself (Needham and Wang 2002) and is an important source of bias.

Figure 1G represents two possible circumstances in which serum PCBs are causally related or correlated with the true exposure/outcome association. If the relation between serum and adipose concentration levels of PCBs is governed by serum lipid levels, then standardization may allow use of one as a proxy for the other.

#### Adjusted model.

In the adjusted model, there is an assumption that PCBs are not standardized for serum lipids, reflecting the absence of an association between lipids and the study outcome. Note that the standardized model is a member of the family of adjusted models.


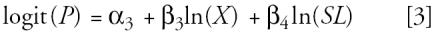


When comparing the lipid component in the standardized model [ln(*X*) − *m* × ln(*SL*)] with the lipid term of the adjusted model [β_4_ ln(*SL*)], equivalent results are produced in that β_4_ is forced to be equal to –*m*. If *m* is set equal to 1, PCBs are divided by serum lipids, as is the case with the standardized model. However, the adjusted model is more flexible than the standardized model and, in general, is applicable under the same set of assumptions.

For Figure 1A, the adjusted model will produce unbiased estimates without regard for the degree of standardization, while the standardized model is conditional on standardization being sufficient. The adjusted model will yield unbiased estimates for Figure 1A, B, D, and F. For Figure 1C, E and H, the adjusted model will yield biased estimates because the adjustment is performed for a variable in the causal pathway; for Figure 1H this bias is to estimates of the total effect due to its partitioning into direct and indirect.

#### Two-stage model.

The two-stage model includes the effects of PCBs and serum lipids on the outcome:


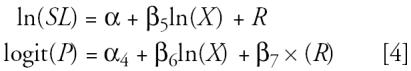


Implications of the two-stage model arise from its relation to the adjusted model. Both the intercept and the beta coefficient in the two-stage model are simple functions of the parameters from the adjusted model and the regression of serum lipids on log PCBs. The coefficient for the residual term, *R*, is precisely that of the adjusted model’s lipids term:


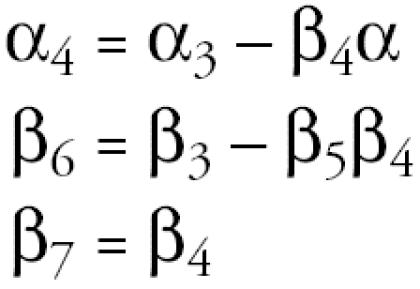


Use of the two-stage model for Figure 1A will result in estimates similar to those produced by the adjusted model, because there is no assumption about an association between PCBs and serum lipids. Therefore, the residuals will be equivalent to the lipid term in the model. The two-stage model may also be used to represent Figure 1F, with an important caveat that the risk estimates now have a different interpretation in that they separate the PCB effect from the lipid effect on the outcome. In some circumstances, the two-stage model will generate unbiased risk estimates for Figure 1B, although they will be inefficient. Similarly, the model may yield unbiased risk estimates for Figure 1C although confounding is not addressed.

The two-stage model is appropriate when it is important to distinguish direct and indirect effects of PCBs (Figure 1H). In this scenario, the effect of serum lipids is an indirect effect via PCBs; their inclusion introduces bias as is the case for the standardized model where assumptions of causality may not be clearly delineated.

### Simulations.

In addition to showing causality in a statistical model, each DAG can be used to guide model selection. We conducted a simulation study to evaluate the utility of various models for various scenarios depicted by DAGs. We used the causal structures they define, assigned lognormal distributions for PCB and serum lipids, and assumed a binomial outcome variable *Y* with *Pr*(*Y* = 1 | PCB, serum lipids). For example, in Figure 1H PCB causes disease *Y* and affects serum lipid (which in turn also affects disease); these associations motivate the model:


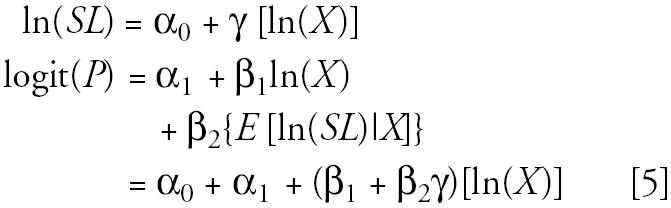


The log odds [logit(*P*(*X*, *SL*)] equals an intercept (α_0_), the prevalence among the unexposed, plus the factor, β_1_+β_2_γ, by which PCB affects the probability of the event. There is no serum lipid term, denoting that there is no linear influence of serum lipid levels.

In Figure 1, the assumptive role of serum lipids is variously *a*) an independent cause, *b*) a dependent cause, *c*) an independent noncause, *d*) a dependent noncause, and *e*) a modifier. *A* represents an unmeasured variable that is an ancestor to both PCB and serum lipids (e.g., fish consumption) that may result in confounding (Hernan 2001, Hernan et al. 2002).

Additionally, we assessed the effects of serum lipid measurement error [ɛ~N(0, σ*_e_*^2^)] with different values of σ*_e_*^2^ and the relation between PCB and serum lipids by varying the strength of their linear relation, α, from the linear regression, *SL* = α_0_ + α*X*.

In these quantitative representations of the DAGs, it is clear that magnitude of effects, error, and bias will be functions of the values chosen for the parameters. We set the independent effect of PCB as a constant (β_lnPCB_ = 0.6 in the logistic regression model), with approximate values taken from the literature (Wolff and Toniolo 1995). In our unpublished data, we observed a significant linear relation between total serum PCBs and serum lipids with a regression coefficient value of approximately 0.3. The values provided for the strength of the linear relation between PCB, and serum lipids represented a very weak association (α = 0.01) to a strong association (α = 2.0).

## Results

Table 1 displays the bias and mean square error for estimates that result from the four statistical models given the underlying causal truths for σ*_e_*^2^ = 1, and α = 0.3. For Figure 1A, which represents PCB and SL as independent causes of the outcome, all models except the standardized produce minimally biased estimates. The standardized model results in a biased underestimate of the PCB effect on outcome. When SL is completely extraneous, as in Figure 1B, bias occurs similar to the previous situation. Figure 1C depicts the effect of PCB acting strictly through SL and is estimated unbiasedly by the two-stage approach. The unadjusted model produces minimal bias. Adjustment for SL results in a large underestimate of effect, as does standardization, though underestimates resulting from standardization are substantially greater (351%). When SL is affected by PCB but does not directly influence the outcome (Figure 1D), standardization is the only modeling approach with substantial bias, underestimating the true effect by nearly 80%, whereas the other models are within 1% of the true effect. In the confounded case, (Figure 1E), only the adjusted model performed well. Lack of adjustment failed to address the confounding by SL, and standardization was not a sufficient method to account for this confounder. In adjusting for serum lipids via the residuals, the two-stage model misattributes the association between PCB and SL as a causal link and results in biased estimates of the effect of interest—the total effect of PCB on risk. Figure 1F represents a noncausal correlation between PCB and SL and, as for Figure 1A, B, and D, produced biased underestimates using the standardized model. Figure 1G is unique among the DAGs in that it posits that serum levels of PCB are dependent on levels in adipose, which are in turn causally related to the outcome. In this situation, standardization functioned optimally; the adjusted model produced similarly unbiased estimates, while neither the unadjusted nor two-stage model worked well. Figure 1H represents a direct and indirect causal link of PCB with outcome. The relation was modeled well by the unadjusted (which estimates total effect) and the two-stage (which separates total into estimated direct and indirect) approaches. Adjustment resulted in a small amount of bias, and standardization produced the most biased estimates in this scenario.

The foregoing results indicate that the standardized and the adjusted models should be compared. With the exception of Figure 1G, the adjusted model produces smaller bias than the standardized model. However, even under conditions ideally suited for the standardized model (Figure 1G: adipose PCB causes both serum PCB per serum lipids and the outcome), the adjusted model yielded a nearly identically unbiased estimate. The two-stage model produced results similar to those of the unadjusted model, though less biased, for Figure 1C, for which serum lipids are in the causal pathway of PCBs and outcome.

### Measurement error.

**Figure 2:**
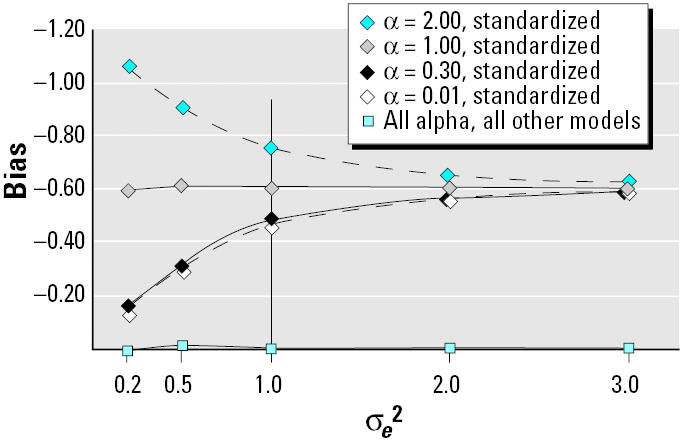
Comparison of bias for standardization versus all other models as a function of measurement error of serum lipids and strength of linear association of PCB with serum lipids for Figure 1A, B, D, and F. Bias for the standardized model was systematically centered on −0.60 (100% underestimation). As measurement error increased, the impact of the strength of the relation between PCB and serum lipid was reduced. None of the other models were sensitive to measurement error under any conditions of the PCB–serum lipid relation. The vertical line at σ*_e_*^2^ = 1 signifies the level used for Table 1.

**Figure 3:**
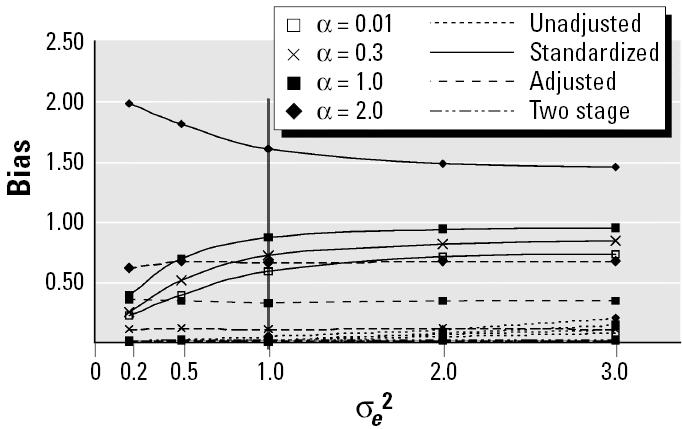
Bias as a function of measurement error of serum lipids and strength of linear association of PCB with serum lipids for Figure 1C, E, and H. The vertical line at σ*_e_*^2^ = 1 signifies the level used for Table 1.

**Figure 4:**
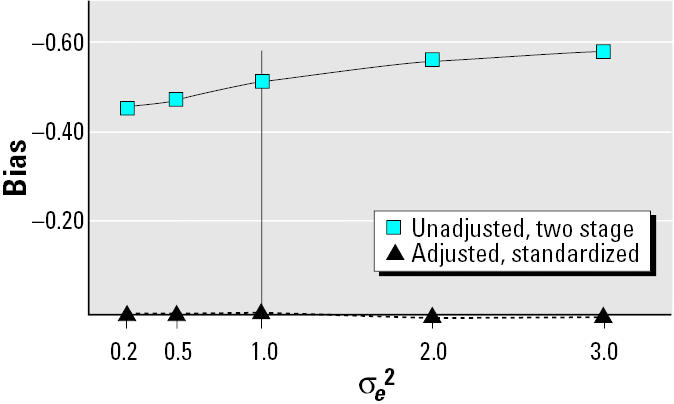
Bias as a function of measurement error of serum lipids and strength of linear association of PCB with serum lipids for Figure 1G. For this causal diagram, the standardized and adjusted models track together and are robust to both measurement error and the strength of the linear relation between PCB and serum lipid. The unadjusted and two-stage models also track together and are somewhat affected by increasing measurement error, although not to changes in the strength of the relation between PCB and serum lipid. The vertical line at σ*_e_*^2^ = 1 signifies the level used for Table 1.

To address the potential for measurement error accompanying quantification of serum lipids, an error term with mean 0 and variance σ*_e_*^2^ was added to the simulated distribution of serum lipids. Figures 2–4 display bias as a function of this measurement error at 4 values of α for each of the models (unadjusted, standardized, adjusted, and two-stage). Bias as a function of σ*_e_*^2^ followed three distinct patterns among the eight DAGs. Figure 2 displays the pattern for Figure 1A, B, D, and F; with increasing measurement error, bias was stable for the unadjusted, adjusted, and two-stage models, staying close to zero. For the standardized model the relation between bias and σ*_e_*^2^ was more complicated; bias increased with measurement error when the relation between PCB and lipids was weak, but at the highest value of α evaluated, bias decreased with measurement error. The value of σ*_e_*^2^ at the inflection point varied from 0.5 for Figure 1F to 3.0 for Figure 1A.

Figure 3 displays the pattern of bias observed when Figure 1C, E, and H depict the truth. Similar to pattern 1, bias for the standardized model varied in a nonlinear manner, increasing for all values of α but the highest (α = 2). The adjusted and two-stage models were essentially robust to measurement error; however, both the unadjusted and adjusted did not always produce unbiased estimates of parameters for all underlying DAGs, especially at different levels of α. A stronger linear relation between PCB and lipids resulted in greater bias in the adjusted model. Bias of estimates produced by the unadjusted model varied slightly with σ*_e_*^2^; for Figure 1C and H bias increased slightly with increasing measurement error (from 0 to 0.1 for 8, from 0 to 0.2 for 3). Increasing measurement error in Figure 1E reduced bias as the strength of the noncausal relation between PCBs and serum lipids was altered by the variance in serum lipids.

Figure 4 displays bias for the four models under the conditions represented by Figure 1G. Both the standardized and adjusted models produced unbiased estimates robust to measurement error, whereas the unadjusted and two-stage models produced biased estimates that were equally prone to measurement error. Changes in the strength of the linear relation between PCB and lipids did not affect bias for any of the four models in this scenario.

## Discussion

We have described and evaluated four statistical models (unadjusted, standardized, adjusted, and two-stage) commonly used to assess the effects of lipophilic environmental contaminants on human health when relying on blood specimens for quantifying toxicant concentrations. Our simulations show that each statistical model has minimal bias for at least the causal truth for which it is ideally suited. Although most models performed well under all but one causal scenario, the standardized model produced large biases for most of the evaluated DAGs. The adjusted model produced only a small bias even for the DAG for which standardization is optimal.

We evaluated basic causal scenarios; the eight DAGs we considered included only two to four factors. When additional factors impact levels of both PCB and serum lipids as well as health outcome risk, the evaluation will be more complex, and the trade-off between statistical efficiency and robustness will be more important. Although the adjusted model produced consistently unbiased estimates, there are circumstances where adjustment (or stratification) is inappropriate and should be avoided. For example, adjustment for a collider (an effect of two or more other variables in the graph) has been demonstrated to bias estimators of effect (Greenland and Brumback 2002; Hernan et al. 2002). Additionally, factors that share a common cause will appear correlated in strata of that common cause. Given an alleged relation between PCB and serum lipids, their adjustment might generate spurious associations if an unmeasured factor is related to both serum lipid levels and the outcome.

A discussion of causality, particularly when regarding estimation of direct and indirect causes, necessitates consideration of counter-factuals. Consistent estimation of a direct or indirect effect require at minimum the absence of unmeasured confounding as well as the assumptions of consistency and the existence of a direct effect (Cole and Hernan 2002; Robins 2003). Estimation of causal effects and their relations to DAGs is intimately tied to the notion of counterfactuals. In reality, when a factor impacts an outcome through both direct and indirect pathways, we cannot observe the direct effect in absence of the indirect effect, and vice versa; their estimation depends on counterfactual comparisons (Robins 2003). A general counterfactual model has been proposed that permits the estimation of total and direct effects of fixed and time-varying exposures in longitudinal studies whether randomized or observational in design (Robins et al. 2000). However, a more detailed discussion is beyond the scope of this paper.

Findings from our simulations demonstrate that statistical models failing to uphold underlying assumptions about causality lead to biased results with implications for the interpretation of effects of exposures on human health end points. We speculate that equivocal findings may arise, at least in part, from the varying laboratory and analytic approaches for specifying serum lipids when using nonfasting blood specimens to estimate risk. Investigators must remember to consider biology, biologic medium, and laboratory methodology when specifying a statistical model and its underlying assumptions appropriate for study.

## Correction

Equation 4 was incorrect in the manuscript originally published online but has been corrected here.

## Figures and Tables

**Table 1:** Percent bias of estimates of effect of PCBs on outcome for evaluated statistical models.

	Percent bias (MSE)*^a^*			
DAG (Figure 1)	Unadjusted	Standardized	Adjusted	Two-stage
A	1.2 (1.26)	–51.3 (10.3)	1.8 (1.28)	1.8 (1.28)
B	–0.8 (1.34)	–75.9 (21.1)	–0.7 (1.35)	–0.7 (1.33)
C	–15.4 (2.78)	–351.3 (161.1)	–99.4 (1.59)	1.1 (2.78)
D	0.4 (1.14)	–79.8 (23.3)	0.8 (1.17)	0.5 (1.14)
E	24.0 (3.37)	–128.8 (60.3)	0.1 (1.39)	27.2 (3.37)
F	–0.4 (1.29)	–85.0 (26.4)	–0.1 (1.41)	–0.3 (1.29)
G	–86.3 (27.0)	–1.0 (1.51)	–1.0 (1.51)	–85.9 (27.0)
H	–11.2 (1.75)	–128.3 (59.7)	–25.4 (3.65)	–8.7 (1.75)

Serum lipid measurement error distributed normally with mean 0, variance 1; α (strength of linear relation between log PCB and log serum lipids) = 0.3; 500 repetitions; *n* = 1,000.

a Mean square error multiplied by 100 for illustration (shown in parentheses).
